# scrm: efficiently simulating long sequences using the approximated coalescent with recombination

**DOI:** 10.1093/bioinformatics/btu861

**Published:** 2015-01-08

**Authors:** Paul R. Staab, Sha Zhu, Dirk Metzler, Gerton Lunter

**Affiliations:** ^1^Department of Biology, Ludwig-Maximilians-Universität München, Planegg-Martinsried, Germany and ^2^Wellcome Trust Centre for Human Genetics, University of Oxford, Oxford OX3 7BN, UK

## Abstract

**Motivation:** Coalescent-based simulation software for genomic sequences allows the efficient *in silico* generation of short- and medium-sized genetic sequences. However, the simulation of genome-size datasets as produced by next-generation sequencing is currently only possible using fairly crude approximations.

**Results:** We present the sequential coalescent with recombination model (SCRM), a new method that efficiently and accurately approximates the coalescent with recombination, closing the gap between current approximations and the exact model. We present an efficient implementation and show that it can simulate genomic-scale datasets with an essentially correct linkage structure.

**Availability and implementation:** The open source implementation scrm is freely available at https://scrm.github.io under the conditions of the GPLv3 license.

**Contact:** staab@bio.lmu.de or gerton.lunter@well.ox.ac.uk.

**Supplementary information:** Supplementary data are available at *Bioinformatics* online.

## 1 Introduction

Coalescent simulation is a valuable tool to investigate population genetic data and the demographic processes that shaped them. Simulation programs based on the coalescent with recombination (CWR) such as ms (Hudson, 2002) support a wide range of evolutionary scenarios and are extremely efficient for short- and medium-sized sequences. As the number of recombination events grows exponentially with increasing sequence length, it is however infeasible to simulate whole chromosomes using these methods. This prevents many methods relying on simulations from being applicable to next-generation sequencing datasets.

In order to resolve this problem, McVean and Cardin (2005) introduced the sequentially Markov coalescence (SMC) model, a method that approximates the CWR by partially ignoring genetic linkage between simulated sites. Subsequently, Marjoram and Wall (2006) proposed a modification of this model, termed SMC’, which improved accuracy. The simulation programs MaCS (Chen *et al*., 2009) and fastsimcoal (Excoffier and Foll, 2011) implement SMC’ and allow rapid simulation of chromosome-sized datasets. However, Eriksson *et al.* (2009) found that the decrease in genetic linkage depends on the simulated evolutionary model, which led them to conclude that SMC’ might not be suitable under certain conditions, and suggested that the effect of the approximation needed to be investigated carefully in each application.

We have developed a novel approximation of the CWR, called the sequential coalescent with recombination model (SCRM). Besides algorithmic optimization, it allows for user-controlled arbitrary precision ranging continuously from SMC’ to the full CWR. We here present an efficient implementation of this model, termed scrm, and show that by using an intermediate approximation level it allows the simulation of sequences of arbitrary length with an essentially correct linkage structure.

## 2 Materials and Methods

SCRM is based on the sequential model for building the ancestral recombination graph (ARG) by Wiuf and Hein (1999). After sampling an initial genealogy at one end of a chromosome, it moves along the sequence and updates the genealogy as recombination events are encountered. The genealogy includes so-called non-local branches, which belong to a previous genealogy but do not carry ancestral material for the current position. These may become important at upstream positions.

Every recombination introduces additional non-local branches, which causes the ARG to grow exponentially along the sequence and makes it impractical to simulate long sequences under the CWR. To resolve this problem, SCRM adds three modifications to the Wiuf–Hein model:

1. SCRM uses a memory efficient tree-based data structure, which encodes recombinations as non-local leaves rather than splits in the graph.
2. Recombination events on non-local branches are postponed until a local tree is affected, and ignored if the local trees remain unchanged. This requires that we account only for local recombinations while moving along the sequence. Non-local branches accumulate the recombination rate until they are targeted by a coalescence. The time until the next recombination event on this branch is then exponentially distributed with the accumulated rate. This modification is similar to a model proposed by Wang *et al.* (2014).
3. The most crucial modification is that we allow to disregard weak linkage over large genomic regions. We do this by removing non-local branches with an accumulated recombination rate above a threshold. As this threshold corresponds to a certain genomic distance to the local tree, this approximation is equivalent to an ‘exact window’ sliding along the sequence. Positions within the window have the same linkage as in the CWR, while positions further apart have reduced linkage. Setting the window size to 0 reduces the algorithm to the SMC’, while a chromosome-sized window recovers the CWR.

### 2.1 Implementation and validation

We have developed scrm, an efficient open-source implementation of SCRM using C++11. Command line in- and output are designed to be compatible with ms, so that scrm can be used as a drop-in replacement. The supported feature set is similar to ms. Additionally, scrm supports samples at different times and variable recombination rates along the sequence. It is optimized for sample sizes of thousands of individuals.

We validated the implementation by comparing exact simulations to ms. No significant deviations were found using ${\text{χ}}^{2}$ and Kolmogorov–Smirnov tests (Supplementary Table S1).

### 2.2 Approximation of Linkage

**Fig. 1.**
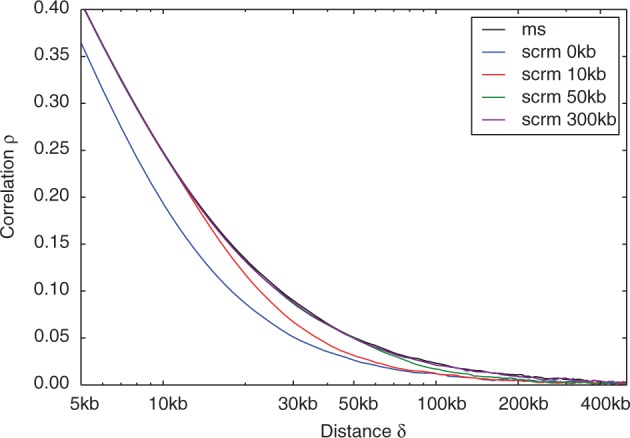
Approximation of genetic linkage. Shown is the correlation of ρ (*y*-axis) of the total local branch length at two sites δ base pairs apart (*x*-axis). The linkage in the CWR (*ms*, options 20 1 -r 4000 10000001 -T) is indicated in black. Results for *scrm* using different exact window sizes (see legend) are indicated in colour

**Fig. 2.**
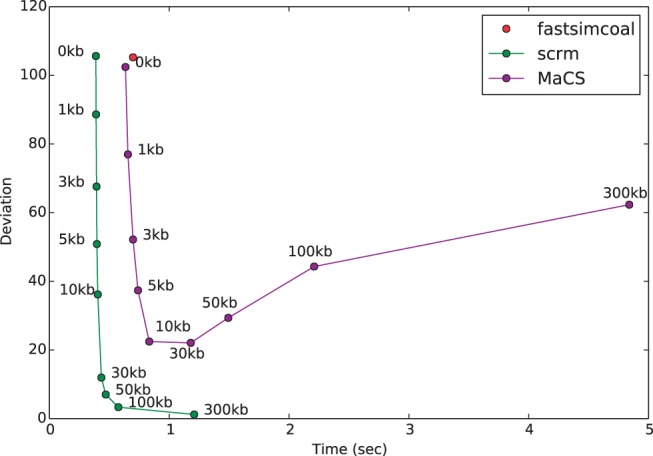
Efficiency for different approximations. Shown is the deviation (*y*-axis) against run-time (*x*-axis) for simulating 10 Mb with a recombination rate of $10^{-8}$ per base per generation. The deviation of the approximation from the correct values is measured as the square root of the area between the ρ−δ correlation curves for the approximate simulated data, and *ms*-generated data (see Fig. 1). For *scrm* and *MaCS* multiple approximation levels are drawn using different exact window sizes or history parameters. The recently published *Cosi2* (Shlyakhter *et al.*, 2014) does not output trees and could not be included in this figure; for a comparison of *Cosi2* and *scrm* using different summary statistics see Supplementary Figure S5

We compared the genetic linkage produced for different levels of approximation by using the correlation of the total local branch length of the genealogy at two sites as a function of their distance (Fig. 1). The ‘exact window’ of scrm is similar to MaCS’s history parameter. However, as MaCS ignores all non-local recombinations, it simulates too much linkage for sites within its history. Consequently, it does not converge to the CWR when reducing the approximation, while scrm does (Fig. 2). In the settings of Figure 2 and using an exact window size of 300 kb, scrm simulates essentially correct linkage across 20 samples with a linear run-time cost of 0.1 s per megabase.

## Funding

This work was supported by the Deutsche Forschungsgemeinschaft [DFG ME 3134/3-2] and the Wellcome Trust [090532/Z/09/Z].

*Conflict of Interest*: none declared.

## Supplementary Material

Supplementary Data
