# Supplementary material for: scrm: efficiently simulating long sequences using the approximated coalescent with recombination
Source: Bioinformatics. 2015 Jan 8;31(10):1680–2. doi: 10.1093/bioinformatics/btu861 (PMC4426833; doi:10.1093/bioinformatics/btu861)
Supplement: Supplementary Data [file supp_31_10_1680__index.html]

scrm: efficiently simulating long sequences using the approximated coalescent with recombination — scrm: efficiently simulating long sequences using the approximated coalescent with recombination — scrm: efficiently simulating long sequences using the approximated coalescent with recombination — Supplementary Data 

# scrm: efficiently simulating long sequences using the approximated coalescent with recombination

## Supplementary Data

files

**Files in this Data Supplement:**

- Supplementary Data - pdf file
