# Supplementary material for: scrm: efficiently simulating long sequences using the approximated coalescent with recombination
Source: Bioinformatics. 2015 Jan 8;31(10):1680–2. doi: 10.1093/bioinformatics/btu861 (PMC4426833; doi:10.1093/bioinformatics/btu861)
Supplement: Supplementary Data [file supp_btu861_scrm-supplement.pdf]

Supplementary material for `scrm`

Contents

1 Algorithm outline 2
1.1 Glossary . . . 2
1.2 Events and rates . . . 2
1.3 Algorithm . . . 4

2 Testing the exact algorithm 6

3 Approximation 8
3.1 Effects of the approximation on summary statistics . . . 8
3.2 Effects of the approximation on linkage . . . 11

4 Efficiency compared to existing software 12
4.1 Linkage comparison of total tree branch length . . . 12
4.2 Total number of mutation comparison between approximated models . . . 12

1 Algorithm outline

The following provides a high level overview of the algorithm SCRM for simulating the Ancestral Recombination Graph under the coalescent with recombination (CWR). The program *scrm* implements this algorithm with modifications to incorporate demographic features such as population size changes, population structure and migration. Most of these modifications are straight-forward manipulation of rates and have only a minor impact on the algorithm.

In general, SCRM alternates between moving along the sequence and moving backwards in time. The current sequence position is denoted with p, and the current time with t. The sequence is assumed to start at position 0 and end at position 1. SCRM simulates a binary tree[1] representing the genealogy of the simulated sequences. This tree changes when moving along the sequence. It consists of local and non-local branches (see section 1.1). The simulated sequences are assumed to be at the "lower" end of the tree, which correspond to recent times. Elements of the tree further in the past are referred to as "upwards" (as in Figure S1).

1.1 Glossary

The following terms are used for describing the algorithm *SCRM*:

local branch	A branch that carries genetic material ancestral to the simulated sequences at the current sequence position p.
non-local branch	A branch that was local for a previous sequence position $p' < p$, but does not carry ancestral material for the current position p.
local recombination	A recombination that occurs on a local branch.
non-local recombination	A recombination that occurs on a non-local branch.
active branch	While going backwards in time, up to two branches can be source of changes to the tree. An active branch can either be coalescing at its top, or be looking for non-local recombinations on itself.
passive branch	A branch that is not active.
local root	The point on the tree where the two oldest local branches merge. There can be a non-local branch on top of the local root, so that the local root is not necessarily the actual root of the tree.
coalescing	If a branch that ends with the root of a tree is coalescing, the top of the branch looks for another branch to merge into. This results in a *Coalescence* event.
looking for non-local recombinations	If a non-local branch is active, it samples whether non-local recombinations are occurring on this branch. This results in a *Non-local recombination* or an *End of branch encountered* event.

1.2 Events and rates

When moving backwards in time, the following events can occur with rates depending on the active nodes or when arriving at a certain time points:

1. Coalescence. Each coalescing branch merges into any branch that exists at the current time with rate one. If two active branches are coalescing, the pair can also merge together with rate one.

2. Non-local recombination. On each branch that is looking for non-local recombinations, recombinations happen with a rate equal to the recombination rate times the sequence length since the branch was last checked for non-local recombinations or became non-local.

[1] There are edge cases in which SCRM produces more than one tree. Technically, SCRM's state space is a sequence of forests (e.g. sets of trees), but for the sake of simplicity, we will ignore these edge cases here.

3. End of branch encountered. If no recombination occurs on a branch looking for recombinations, an end of branch event is created at the end of the branch.

4. Time of local root reached. When time t is less than the time of the local root t_{MRCA}, one branch is active. When $t = t_{MRCA}$, this event additionally activates the branch above the local root.

1.3 Algorithm

1. *"Build an initial tree"*

   1.1 Initialize the position $p = 0$ at one end of the sequence.

   1.2 Build an initial tree according to Kingman's coalescent.

2. *"Move along the sequence to the next local recombination"*

   2.1 Sample the sequence length s until the next local recombination occurs according to an exponential distribution with rate equal to the length of the current local tree.

   2.2 If $p + s > 1$, the algorithm has reach the end of the sequence and is finished. Otherwise, move along the sequence to the local recombination, e.g. $p = p + s$.

   2.3 Sample the time t_{rec} and the branch b_{rec} of the recombination event, which occurs uniformly on the local tree.

3. *"Modify the tree"*

   3.1 Remove the subtree below the local recombination from the tree, and make it a separate tree. Mark the branch above the root of the new tree as the only active branch. Mark every branch above b_{rec} that has not at least one simulated sequence below it as non-local.

   3.2 Set the time t to the time of the recombination t_{rec}.

   3.3 Calculate rates for all possible events (see "Events and rates"). Sample the time length t_{next} until the next event happens according to the rates. Increase the time $t = t + t_{next}$. Continue depending on the type of the event:

   - *Coalescence*. The merge is implemented. If the target branch is
     - a local branch: Modification of tree is complete[2]. Continue with 2.1.
     - the other coalescing node: Modification of tree is complete. Continue with 2.1.
     - a non-local branch: The coalescing branch is no longer active. The target branches becomes active, and starts looking for postponed non-local recombinations on itself. Continue to go back in time, i.e. with 3.3.
   - *Non-local recombination*: The branch becomes passive and the part of the branch below the recombination event is marked as local. The subtree below the recombination is removed from the tree and becomes a separate tree. The root of this tree becomes active and starts coalescing. Continue with 3.3.
   - *End of branch encountered*: The branch is marked as local and not active. If the branch
     - ends at the root of a tree: A new branch is created above the root, marked as active and starts coalescing. Continue with 3.3.
     - leads to a local branch: Modification of tree is complete[2]. Continue with 2.1.
     - leads to a non-local branch: This branch becomes active and starts looking for recombinations. Continue with 3.3.
   - *Time of local root reached*. If there is
     - no branch above the local root: Create a new branch above the root, mark it as active and coalescing.
     - a (necessarily non-local) branch: This branch becomes active and starts looking for non-local recombinations.

   Continue with 3.3, now with two active branches.

[2]Local branches exists only at times more recent than the local root, where only one branch is active. Hence, if an active branch encounters a local branch, it was the only active branch, and the modification of the tree is complete.

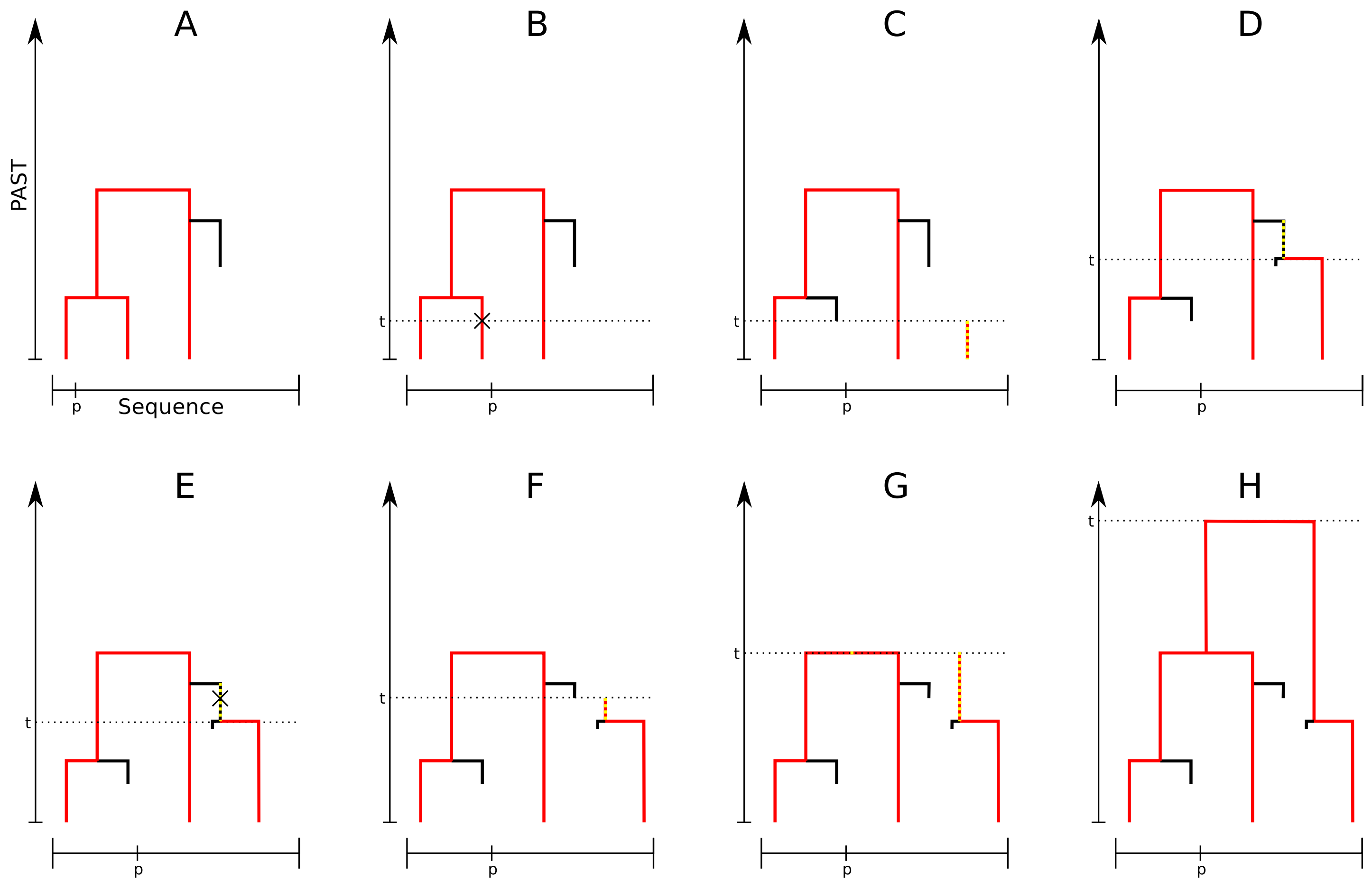

Figure S1: Example of the SCRM algorithm. Red denotes local branches, black non-local one. Active branches are yellow dotted. From (A) to (B) the algorithm moves along the sequence until a recombination occurs (black cross). The recombination is implemented (Step 3.1), which produces (C). The active branch is now *coalescing* and coalesces into the non-local branch above (D). The active branch is now looking for non-local recombinations, one of which occurs in (E). The recombination is implemented (F), and the active branch is *coalescing* again. In (G) the time of the local root reached event occurs. Both active branches are now *coalescing* and coalescent in (G). Now, Step 3 is finished and the algorithm continues with Step 2.

2 Testing the exact algorithm

To verify that both our algorithm and its implementation work correctly, we compared samples produced under the coalescent with recombination to ones produced by `scrm` without approximation. For the comparison, we selected six demographic models (Table S2) and simulated 10^5 samples with both `ms`[3] and `scrm` for each model. We compared the distribution of commonly used summary statistics including the time to the most recent common ancestor (TMRCA), total branch lengths, the number of mutations and recombinations, average pairwise differences, Fay and Wu's θ_H (Fay and Wu, 2000), difference (denoted by H) between θ_H and average pairwise differences, and Tajima's D (Tajima, 1989) using Kolmogorov-Smirnov- and χ^2-tests.

In all models the summary statistic's distributions seemed to be virtually identical. We tested for difference in the distributions Kolmogorov-Smirnov- and χ^2-tests. None of the test found a significant difference at a 95%-confidence level (see Table S1).

Summary statistics of	Case 1	Case 2	Case 3	Case 4	Case 5	Case 6
TMRCA	0.6062	0.9011	0.09087	0.6815	0.511	0.4556
Total branch lengths	0.8376	0.7519	0.4658	0.7805	0.3162	0.8959
Pairwise differences	0.3820	0.08215	0.5543	0.2587	0.9489	0.8711
Theta H	0.6777	0.1691	0.3611	0.1741	0.7944	0.9689
H	0.5912	0.9415	0.2681	0.8797	0.511	0.9489
Tajima D	0.8214	0.6249	0.6062	0.4227	0.2173	0.9037

Table S1: p-values of the summary statistics, calculated by `R`-function `ks.test`.

[3]For the comparison, we modified `ms` to print times in the Newick trees rounded to six (instead of three) decimal places.

Case	Population Structure (from the present to the past)	Samples
1	A split model of two populations (A and B) at generation $3 \times 4N_e$. Global population size change at generation $0.4 \times 4N_e$ and $1 \times 4N_e$ to $10.01 \times N_e$ and $0.01 \times N_e$ respectively. A population bottleneck is presented in population B at time $0.25 \times 4N_e$ with the size of $0.2 \times N_e$.	Ten haplotypes are sampled from two populations: two from population A and eight individuals from population B.
	`ms 10 100000 -t 10 -r 10 100000 -I 2 2 8 -eN 0.4 10.01 -eN 1 0.01 -en 0.25 2 0.2 -ej 3 2 1 -T -L`<br>`scrm 10 100000 -t 10 -r 10 100000 -I 2 2 8 -eN 0.4 10.01 -eN 1 0.01 -en 0.25 2 0.2 -ej 3 2 1 -T -L`	
2	A split model of three populations: The first population split between A and B is at generation $0.7 \times 4N_e$; the second population split happens at generation $1 \times 4N_e$, with the rise of population C; Global population size changes at time $0.8 \times 4N_e$, with an increase to $15 \times N_e$. Symmetrical migration is presented at all times, with the rate of 5.	Fifteen haplotypes are sampled in total: ten samples from population A, four samples from population B, and one sample from population C.
	`ms 15 100000 -t 10 -r 10 100000 -I 3 10 4 1 -ma x 5.0 5.0 5.0 x 5.0 5.0 5.0 x -eN 0.8 15 -ej .7 2 1 -ej 1 3 1 -T -L`<br>`scrm 15 100000 -t 10 -r 10 100000 -I 3 10 4 1 -ma x 5.0 5.0 5.0 x 5.0 5.0 5.0 x -eN 0.8 15 -ej .7 2 1 -ej 1 3 1 -T -L`	
3	Similar population structure as Case 2: The first population separation is the same as Case 2; the second population split happens at generation $4 \times 4N_e$, with the rise of population C; Global population size change at generation $1 \times 4N_e$ and $3 \times 4N_e$ to $0.1 \times N_e$ and $10 \times N_e$ respectively. Migration rates between populations A, B and C are presented in $\begin{bmatrix} 0 & 1.0 & 2.0 \\ 3.0 & 0 & 4.0 \\ 5.0 & 6.0 & 0 \end{bmatrix}$.	The same as Case 2.
	`ms 15 100000 -t 10 -r 10 100000 -I 3 10 4 1 -ma x 1.0 2.0 3.0 x 4.0 5.0 6.0 x -eN 1 .1 -eN 3 10 -ej .7 2 1 -ej 4 3 1 -T -L`<br>`scrm 15 100000 -t 10 -r 10 100000 -I 3 10 4 1 -ma x 1.0 2.0 3.0 x 4.0 5.0 6.0 x -eN 1 .1 -eN 3 10 -ej .7 2 1 -ej 4 3 1 -T -L`	
4	Island-migration model of two populations with symmetrical migration rate of 5.	Two haplotypes are sampled from each population
	`ms 4 100000 -t 10 -r 10 100000 -I 2 2 2 5.0 -T`<br>`scrm 4 100000 -t 10 -r 10 100000 -I 2 2 2 -ma x 5.0 5.0 x -T -L`	
5	Island-migration model of two populations with migration asymmetrical migration rate: 10 from B to A; 5 from A to B.	Two haplotypes are sampled from each population
	`ms 4 100000 -t 10 -r 10 100000 -I 2 2 2 -ma x 10.0 5.0 x -T`<br>`scrm 4 100000 -t 10 -r 10 100000 -I 2 2 2 -ma x 10.0 5.0 x -T -L`	
6	Island-migration model of three populations with symmetrical migration rate of 5.	The same as Case 2.
	`ms 15 100000 -t 10 -r 10 100000 -I 3 10 4 1 10.0 -T`<br>`scrm 15 100000 -t 10 -r 10 100000 -I 3 10 4 1 10.0 -T -L`	

Table S2: Demographic models used when checking `scrm` for exact ARG simulations. All sequences have lengths of 100kb, with recombination and mutation rates both equal to 10.

3 Approximation

When building the ARG using SCRM, the effect that non-local branches have on the genealogy after the next recombination events decays with genetic distance. As we move on along the sequence, non-local branches that are not hit by other coalescing branches accumulate a high recombination rate. If such a branch is hit while incorporating the next recombination, the next non-local recombination on this branch will likely occur close to the coalescence point. As the algorithm continues coalescing above the non-local recombination, it will be in a similar state as it was before hitting the branch. Hence non-local branches with high accumulated recombination rates contribute only minor 'jumps' backwards in time to the algorithm. This observation is the key reason that linkage between sites decays with increasing genetic distance in the ARG.

Different to the WH-Algorithm, SCRM is able to easily identify branches with a high accumulated recombination rate. We use this to introduce an approximation by removing branches that are not hit by coalescing branches while moving on the sequence for more than l bases, where l is an arbitrary threshold. This results in a sliding window of length l, within which SCRM produces the ARG, while it ignores some genetic linkage to positions outside of this window. If the threshold l is equal to sequence length, SCRM produces the exact ARG; SCRM is identical to the SMC' model if $l = 0$.

3.1 Effects of the approximation on summary statistics

We additionally compared the results of simulations of `ms` and `scrm` with approximation windows sizes of $l = 0kB$, $l = 10kB$, $l = 50kB$ and $l = 300kB$ using the same procedure as when comparing the exact algorithms above. In addition, we also calculated average TMRCA across the simulated sequence and compared the empirical distribution of its first four moments to `ms` (Figure S3). Simulated data were genereated by the following command:

```
ms 6 1 -T -t 8000 -r 4000 10000001 -seed ${i} ${i} ${i}
```

and

```
scrm 6 1 -T -t 8000 -r 4000 10000001 -seed ${i} -l ${window}
```

where `${i}` are ranged from 1 to 100000, and `${window}` takes value 0, 10000, 50000, and 300000.

We tested how the approximation parameter affects the simulation output, and the distribution of common summary statistics (Figures S2). A clear difference in the distribution of summary statistics was found when comparing the SMC' to the CWR. As expected the difference decreases when increasing the window size. Some summary statistics appeared less sensitive to the approximation parameters than the others. For example, there is no significant difference in the distribution of Tajima'D or H when the approximation parameter is greater than 50kb. When using `scrm` in with a window length of 300kb or in exact mode, none of the summary statistics showed a significant difference from `ms`. A similar pattern is observed when looking at the distribution of average TMRCAs (Figures S3).

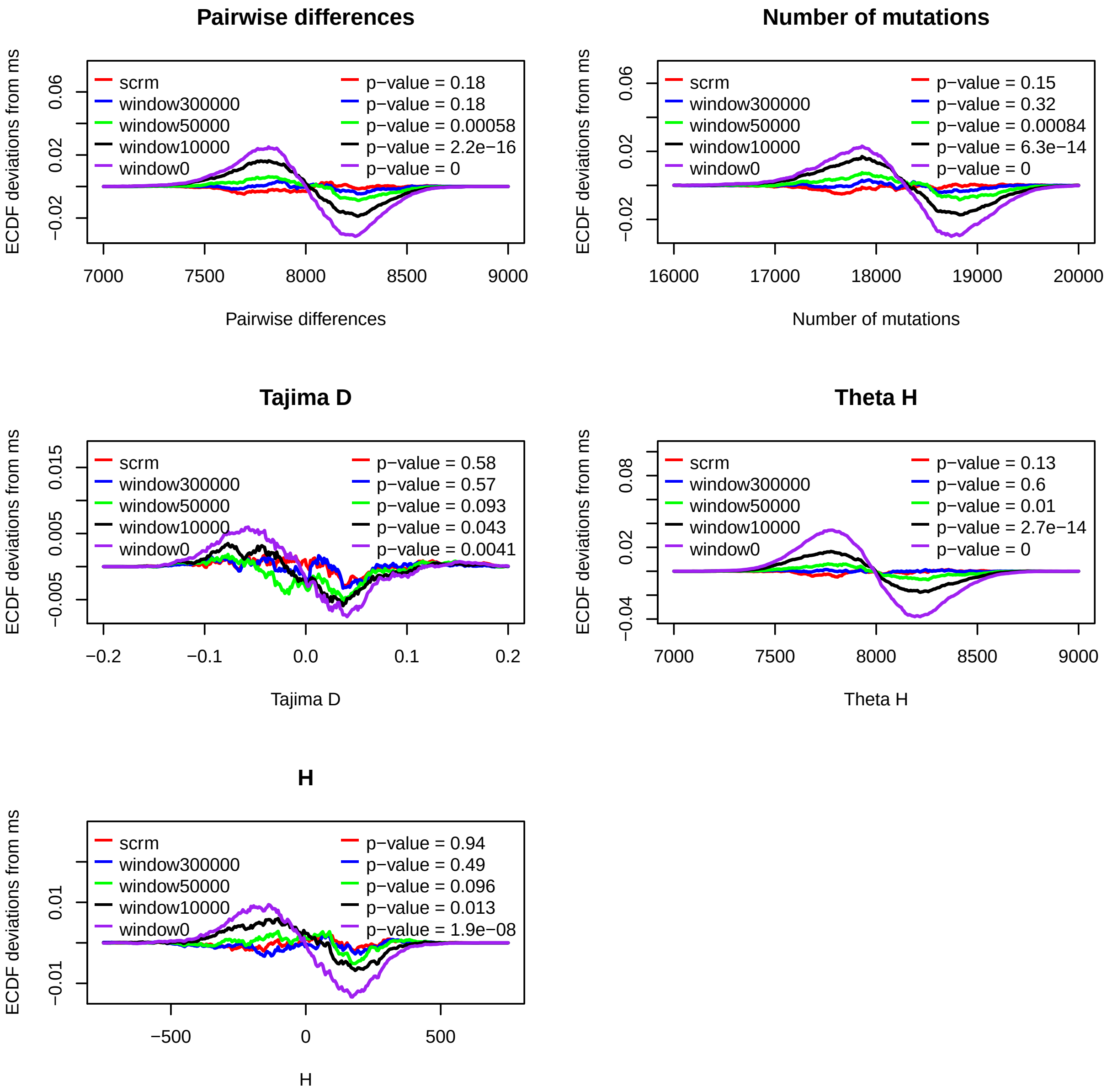

Figure S2: Deviation of the empirical distribution of different summary statistics from `ms`. The p-values are calculated by `R`-function `ks.test`.

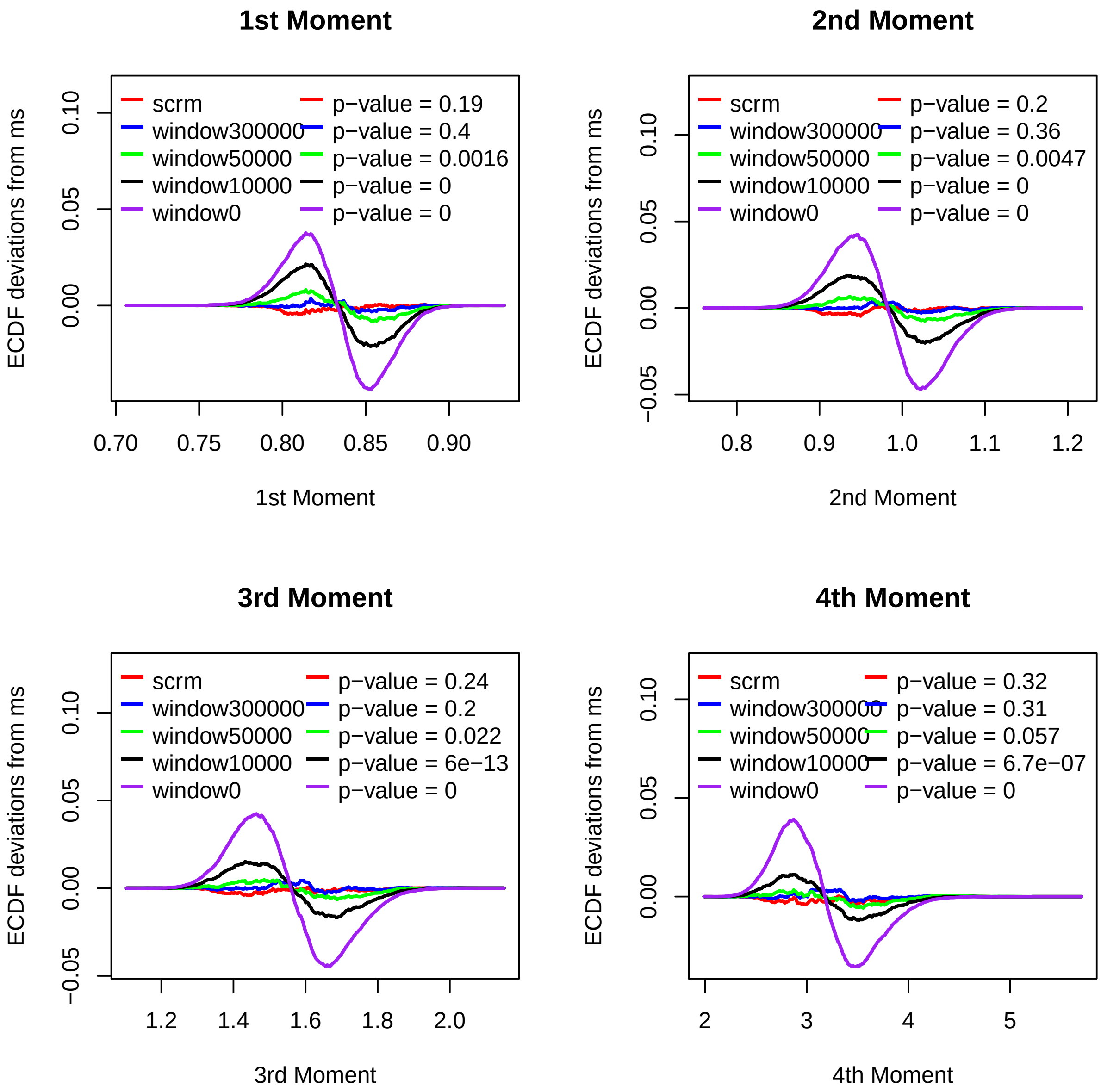

Figure S3: Deviation of the empirical distribution of the first four moments of the average TMRCA from `ms`. The p-values are calculated by `R`-function `ks.test`.

3.2 Effects of the approximation on linkage

Similar to the analysis of Eriksson *et al.* (2009), we used the correlation of the total branch length between two sites to quantify the difference in genetic linkage between the CWR and it's approximations produced by `scrm`. Let $\rho(\delta)$ denote the branch length correlation of two sites that are δ base pairs apart. We investigated how $\rho(\delta)$ differs between sequences generated with `ms` using the exact ARG and with `scrm` using the approximated ARG for two loci as a function of their distance δ for different values of the approximation parameter l.

Let $L(i)$ denote the total branch length of the local genealogy at the site i, we define the branch length correlation between site i and $i+\delta$ as

$$\rho(\delta) = \frac{E[(L(i) - E[L]) \cdot (L(i+\delta) - E[L])]}{E[(L(i) - E[L]) \cdot (L(i) - E[L])]}, \qquad (1)$$

where we estimated the expected total branch length $E[L]$ empirically using simulation with `ms`. The genealogies were generated by the following command:

```
ms 20 1 -T -r 4000 10000001 -seed ${i} ${i} ${i}
```

and

```
scrm 20 1 -T -r 4000 10000001 -seed ${i} -l ${window}
```

where `${i}` are ranged from 1 to 1000, and `${window}` takes value 0, 1000, 3000, 5000, 10000, 30000, 50000, 100000 and 300000.

We found that when using the exact window approach to approximate the CWR, the correct LD is procured as long as both sites are inside the exact window. The linkage dropped rapidly if δ became larger than the window length l. As linkage decreased with distance between the sites, the maximal possible error introduced by the approximation is also decreasing if l becomes larger. The correlation ρ decreased surprisingly slowly. We still found measurable correlation for sites 100kb apart, which is equivalent to an average of 140 recombination events (Figure S4).

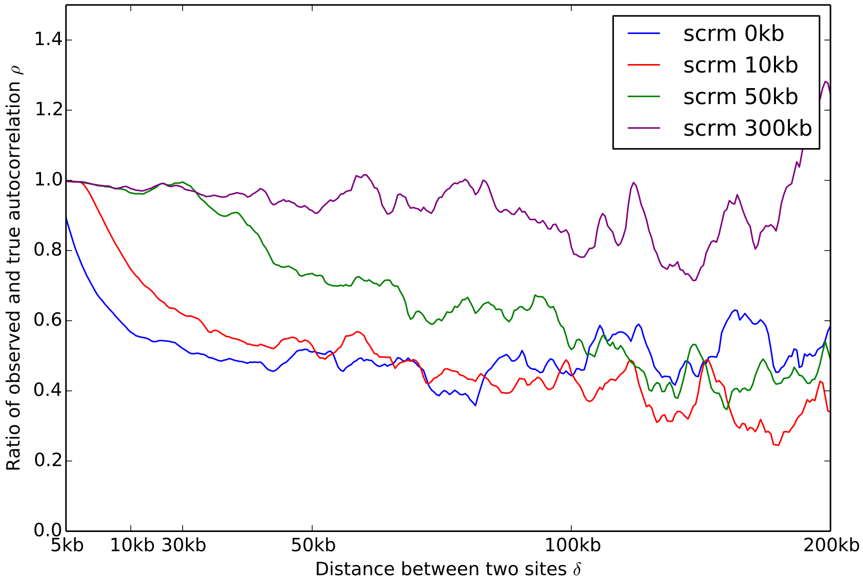

Figure S4: Ratio of observed and true autocorrelations in total branch length linkage of `scrm`, where the true autocorrelations are estimated by `ms`. The increasing variation for higher values of δ is due to the decreasing total linkage between sites.

4 Efficiency compared to existing software

4.1 Linkage comparison of total tree branch length

We measured how efficiently `scrm` performs in comparison to the programs `fastsimcoal` and `MaCS`. Similarly to the comparison in section 3.2, we simulated 20 haplotypes with length of 10 Mb, using a recombination rate of 10^{-8} under a panmictic model with each program, measured the run-time and used the integrated difference of $\rho(\delta)$ to `ms` to measure the accuracy of the programs. For software that offers a variable approximation level, we use different approximation parameters. We compared run-time and difference in linkage to the ARG for the programs `fastsimcoal`, `MaCS` and `scrm`, which all approximate the ARG. When implementing the SMC' model, all programs produced less linkage than the ARG. Simulations with `scrm` appeared to be more efficient than `MaCS` and `fastsimcoal`, and about 50 times faster than `ms`. When `MaCS`' history parameter and `scrm`'s exact window were used, the linkage became more similar to the ARG. When used with a large history, results from `MaCS` showed more linkage than the ARG, while `scrm` approached the correct value, see Figure 2 in the main article.

4.2 Total number of mutation comparison between approximated models

The program `Cosi2` (Shlyakhter *et al.*, 2014) is an efficient simulator which generates the CWR backwards in time instead of following a sequential model. It simulates the CWR extremely efficiently for sequences on an order of several megabases, and also has an approximation feature. Unfortunately, `cosi2` seems to be not capable of printing the simulated trees. Hence, it was not possible to compare `cosi2`'s total branch length linkage with the rest of the programs in section 4.1. Instead, we used the distribution of the number of mutations for a separate comparison (Figure S5).

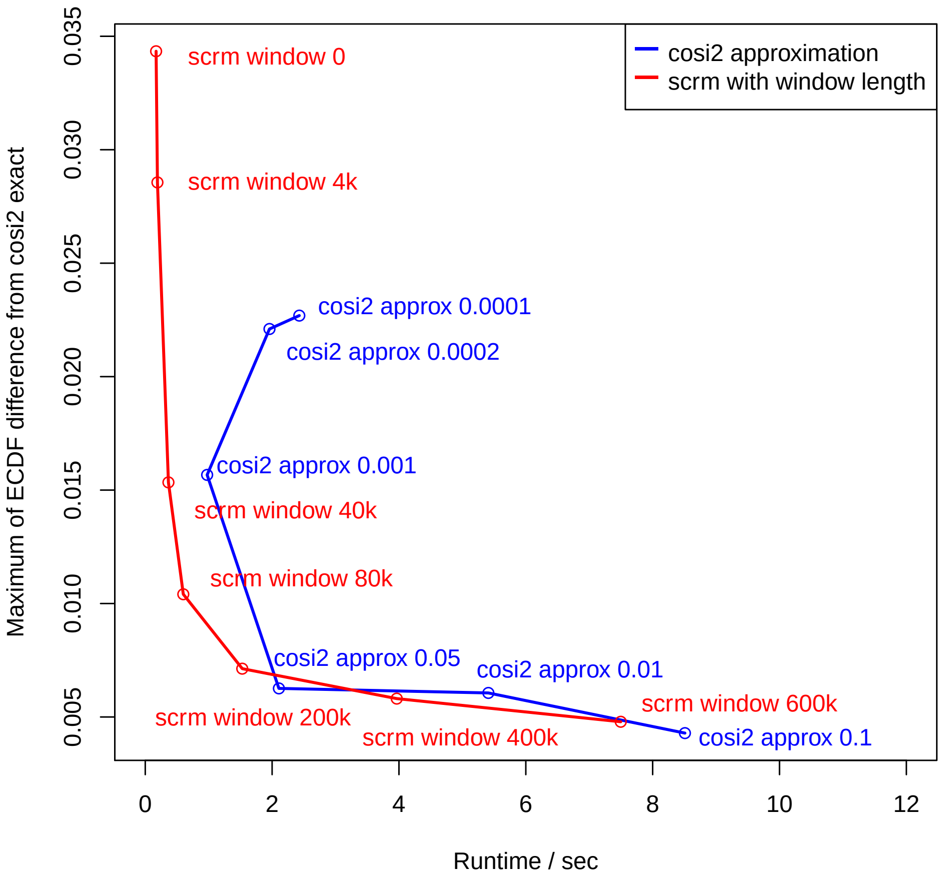

Figure S5: Efficiency of the approximations of `scrm` and `Cosi2`. The runtime (x-axis) for simulating 40Mb with a recombination and mutation rate of 10^{-8} using the indicated approximation parameter is drawn. For the y-axis, the empirical cumulative density function (ECDF) of the number of mutations is calculated from 10^6 simulations (as in Figure S2) and the maximal difference of this function from the ECDF of the exact simulation with `Cosi2` is drawn. We use `Cosi2` as reference in this figure as `ms` can not simulate the used scenario. We suppose that the minimal difference obtained by both programs reflects the stochasticity of obtaining the ECDF from a finite sample.

References

Eriksson, A., Mahjani, B., and Mehlig, B. (2009). Sequential markov coalescent algorithms for population models with demographic structure. *Theoretical Population Biology*, **76**(2), 84–91.

Fay, J. C. and Wu, C.-I. (2000). Hitchhiking under positive darwinian selection. *Genetics*, **155**(3), 1405–1413.

Shlyakhter, I., Sabeti, P. C., and Schaffner, S. F. (2014). Cosi2: an efficient simulator of exact and approximate coalescent with selection. *Bioinformatics*, **30**(23), 3427–3429.

Tajima, F. (1989). Statistical method for testing the neutral mutation hypothesis by dna polymorphism. *Genetics*, **123**(3), 585–95.
